# 2-(4-Chloro­phen­yl)-3-methyl­sulfanyl-5-phenyl-1-benzofuran

**DOI:** 10.1107/S1600536810008706

**Published:** 2010-03-13

**Authors:** Hong Dae Choi, Pil Ja Seo, Byeng Wha Son, Uk Lee

**Affiliations:** a Department of Chemistry, Dongeui University, San 24 Kaya-dong Busanjin-gu, Busan 614-714, Republic of Korea; b Department of Chemistry, Pukyong National University, 599-1 Daeyeon 3-dong, Nam-gu, Busan 608-737, Republic of Korea

## Abstract

In the title compound, C_21_H_15_ClOS, the 4-chloro­phenyl ring is rotated out of the benzofuran plane, making a dihedral angle of 21.50 (6)°. The dihedral angle between the 5-phenyl ring and the benzofuran plane is 29.39 (6)°. The crystal studied was an inversion twin with a 0.65 (7):0.35 (6) domain ratio.

## Related literature

For the crystal structures of similar benzofuran derivatives, see: Choi, *et al.* (2009 ▶, 2010 ▶). For the pharmacological activity of benzofuran compounds, see: Aslam *et al.* (2006 ▶); Galal *et al.* (2009 ▶); Khan *et al.* (2005 ▶). For natural products with benzofuran rings, see: Akgul & Anil (2003 ▶); Soekamto *et al.* (2003 ▶).
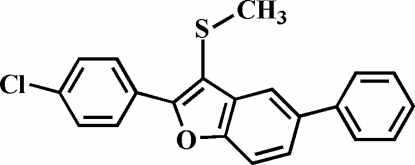


## Experimental

### 

#### Crystal data

C_21_H_15_ClOS
*M* *_r_* = 350.84
Monoclinic, 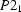

*a* = 10.921 (1) Å
*b* = 7.2225 (8) Å
*c* = 11.740 (1) Å
β = 115.132 (6)°
*V* = 838.35 (14) Å^3^
*Z* = 2
Mo *K*α radiation
μ = 0.36 mm^−1^
*T* = 173 K
0.27 × 0.15 × 0.14 mm

#### Data collection

Bruker SMART APEXII CCD diffractometer
Absorption correction: multi-scan (*SADABS*; Bruker, 2009 ▶) *T*_min_ = 0.911, *T*_max_ = 0.951
7754 measured reflections
3571 independent reflections
3376 reflections with *I* > 2σ(*I*)
*R*_int_ = 0.031

#### Refinement

*R*[*F* ^2^ > 2σ(*F* ^2^)] = 0.035
*wR*(*F* ^2^) = 0.093
*S* = 1.05
3571 reflections
219 parameters
1 restraint
H-atom parameters constrained
Δρ_max_ = 0.29 e Å^−3^
Δρ_min_ = −0.23 e Å^−3^
Absolute structure: Flack (1983 ▶), 1505 Friedel pairs
Flack parameter: 0.35 (6)

### 

Data collection: *APEX2* (Bruker, 2009 ▶); cell refinement: *SAINT* (Bruker, 2009 ▶); data reduction: *SAINT*; program(s) used to solve structure: *SHELXS97* (Sheldrick, 2008 ▶); program(s) used to refine structure: *SHELXL97* (Sheldrick, 2008 ▶); molecular graphics: *ORTEP-3* (Farrugia, 1997 ▶) and *DIAMOND* (Brandenburg, 1998 ▶); software used to prepare material for publication: *SHELXL97*.

## Supplementary Material

Crystal structure: contains datablocks global, I. DOI: 10.1107/S1600536810008706/fl2295sup1.cif

Structure factors: contains datablocks I. DOI: 10.1107/S1600536810008706/fl2295Isup2.hkl

Additional supplementary materials:  crystallographic information; 3D view; checkCIF report

## Figures and Tables

**Fig. 1.**
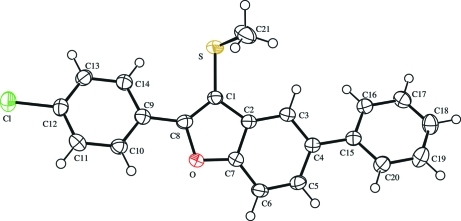
The molecular structure of the title compound with the atom numbering scheme. Displacement ellipsoids are drawn at the 50 % probability level. H atoms are presented as a small spheres of arbitrary radius.

## supplementary crystallographic information

### Comment

Compounds containing a benzofuran moiety show diverse pharmacological activities such as antifungal (Aslam *et al.*, 2006), antitumor and antiviral (Galal *et al.*, 2009), and antimicrobial (Khan *et al.*, 2005) properties. These compounds occur widely in nature (Akgul & Anil, 2003; Soekamto *et al.*, 2003). As a part of our ongoing studies of the effect of side chain substituents on the solid state structures of 3-alkylsulfanyl-2-(4-fluorophenyl)-5-phenyl-1-benzofuran analogues (Choi *et al.*, 2009, 2010), we report the crystal structure of the title compound (Fig. 1).

The title compound crystallizes as the monoclinic space group P21. The crystal studied was an inversion twin with a 0.65 (7) : 0.35 (6) domain ratio. The benzofuran unit is essentially planar, with a mean deviation of 0.012 (2) Å from the least-squares plane defined by the nine constituent atoms. The dihedral angle formed by the benzofuran plane and the 4-fluorophenyl ring is 21.50 (6)°. The dihedral angle between the 5-phenyl ring and the benzofuran plane is 29.39 (6)°. No unusually close intermolecular interactions were found.

### Experimental

Zinc chloride (273 mg, 2.0 mmol) was added to a stirred solution of 4-phenylphenol (340 mg, 2.0 mmol) and 2-chloro-2-methylsulfanyl-4'-chloroacetophenone (470 mg, 2.0 mmol) in dichloromethane (30 ml) at room temperature, and stirring was continued at the same temperature for 40 min. The reaction was quenched by the addition of water and the organic layer separated, dried over magnesium sulfate, filtered and concentrated at reduced pressure. The residue was purified by column chromatography (carbon tetrachloride) to afford the title compound as a colorless solid [yield 51%, m.p. 420–421 K; *R*_f_ = 0.63 (carbon tetrachloride)]. Single crystals suitable for X-ray diffraction were prepared by evaporation of a solution of the title compound in carbon tetrachloride at room temperature.

### Refinement

The reported Flack parameter was obtained by TWIN/BASF procedure in SHELXL97-2 (Sheldrick, 2008). All H atoms were geometrically positioned and refined using a riding model, with C–H = 0.95 Å for aryl and 0.98 Å for methyl H atoms. *U*_iso_(H) = 1.2*U*_eq_(C) for aryl and 1.5*U*_eq_(C) for methyl H atoms.

### Crystal data

**Table:**

C_21_H_15_ClOS	*F*(000) = 364
*M**_r_* = 350.84	*D*_x_ = 1.390 Mg m^−^^3^
Monoclinic, *P*2_1_	Mo *K*α radiation, λ = 0.71073 Å
Hall symbol: P 2yb	Cell parameters from 4886 reflections
*a* = 10.921 (1) Å	θ = 3.4–27.4°
*b* = 7.2225 (8) Å	µ = 0.36 mm^−^^1^
*c* = 11.740 (1) Å	*T* = 173 K
β = 115.132 (6)°	Block, colourless
*V* = 838.35 (14) Å^3^	0.27 × 0.15 × 0.14 mm
*Z* = 2	

### Data collection

**Table:**

Bruker SMART APEXII CCD diffractometer	3571 independent reflections
Radiation source: Rotating Anode	3376 reflections with *I* > 2σ(*I*)
Bruker HELIOS graded multilayer optics	*R*_int_ = 0.031
Detector resolution: 10.0 pixels mm^-1^	θ_max_ = 27.4°, θ_min_ = 1.9°
φ and ω scans	*h* = −14→13
Absorption correction: multi-scan (*SADABS*; Bruker, 2009)	*k* = −9→8
*T*_min_ = 0.911, *T*_max_ = 0.951	*l* = −15→15
7754 measured reflections	

### Refinement

**Table:**

Refinement on *F*^2^	Secondary atom site location: difference Fourier map
Least-squares matrix: full	Hydrogen site location: difference Fourier map
*R*[*F*^2^ > 2σ(*F*^2^)] = 0.035	H-atom parameters constrained
*wR*(*F*^2^) = 0.093	*w* = 1/[σ^2^(*F*_o_^2^) + (0.051*P*)^2^ + 0.0803*P*] where *P* = (*F*_o_^2^ + 2*F*_c_^2^)/3
*S* = 1.05	(Δ/σ)_max_ < 0.001
3571 reflections	Δρ_max_ = 0.28 e Å^−^^3^
219 parameters	Δρ_min_ = −0.23 e Å^−^^3^
1 restraint	Absolute structure: Flack (1983), 1505 Friedel pairs
Primary atom site location: structure-invariant direct methods	Flack parameter: 0.35 (6)

### Special details

**Table:**

Geometry. All esds (except the esd in the dihedral angle between two l.s. planes) are estimated using the full covariance matrix. The cell esds are taken into account individually in the estimation of esds in distances, angles and torsion angles; correlations between esds in cell parameters are only used when they are defined by crystal symmetry. An approximate (isotropic) treatment of cell esds is used for estimating esds involving l.s. planes.
Refinement. Refinement of F^2^ against ALL reflections. The weighted R-factor wR and goodness of fit S are based on F^2^, conventional R-factors R are based on F, with F set to zero for negative F^2^. The threshold expression of F^2^ > 2sigma(F^2^) is used only for calculating R-factors(gt) etc. and is not relevant to the choice of reflections for refinement. R-factors based on F^2^ are statistically about twice as large as those based on F, and R- factors based on ALL data will be even larger.

### Fractional atomic coordinates and isotropic or equivalent isotropic displacement parameters (Å^2^)

**Table:**

	*x*	*y*	*z*	*U*_iso_*/*U*_eq_	
Cl	−0.26159 (5)	0.53997 (9)	−0.01679 (5)	0.04418 (14)	
S	0.45654 (5)	0.50246 (9)	0.13481 (4)	0.04300 (16)	
O	0.37078 (12)	0.5417 (2)	0.43085 (10)	0.0297 (3)	
C1	0.44798 (18)	0.5179 (3)	0.28020 (15)	0.0289 (4)	
C2	0.56317 (17)	0.5235 (3)	0.40109 (15)	0.0267 (3)	
C3	0.70372 (18)	0.5202 (3)	0.44147 (15)	0.0276 (4)	
H3	0.7412	0.5078	0.3822	0.033*	
C4	0.78803 (17)	0.5353 (3)	0.56955 (15)	0.0270 (3)	
C5	0.72841 (19)	0.5488 (3)	0.65544 (16)	0.0311 (4)	
H5	0.7859	0.5565	0.7428	0.037*	
C6	0.58971 (19)	0.5512 (3)	0.61716 (16)	0.0321 (4)	
H6	0.5513	0.5607	0.6759	0.039*	
C7	0.51007 (18)	0.5391 (3)	0.48929 (15)	0.0287 (3)	
C8	0.33570 (18)	0.5302 (3)	0.30249 (15)	0.0286 (4)	
C9	0.19012 (18)	0.5351 (3)	0.22349 (15)	0.0281 (3)	
C10	0.1017 (2)	0.6099 (3)	0.26900 (18)	0.0297 (4)	
H10	0.1372	0.6584	0.3520	0.036*	
C11	−0.0366 (2)	0.6149 (3)	0.19605 (19)	0.0329 (4)	
H11	−0.0955	0.6672	0.2281	0.039*	
C12	−0.08758 (18)	0.5422 (3)	0.07541 (16)	0.0304 (4)	
C13	−0.0032 (2)	0.4675 (3)	0.02688 (17)	0.0335 (4)	
H13	−0.0399	0.4188	−0.0561	0.040*	
C14	0.1356 (2)	0.4641 (3)	0.10026 (18)	0.0325 (4)	
H14	0.1939	0.4135	0.0670	0.039*	
C15	0.93761 (17)	0.5412 (3)	0.61447 (15)	0.0271 (3)	
C16	1.0017 (2)	0.4543 (3)	0.54744 (18)	0.0316 (4)	
H16	0.9492	0.3851	0.4739	0.038*	
C17	1.1406 (2)	0.4677 (3)	0.58677 (19)	0.0364 (4)	
H17	1.1821	0.4085	0.5399	0.044*	
C18	1.2186 (2)	0.5668 (3)	0.6939 (2)	0.0413 (5)	
H18	1.3134	0.5781	0.7198	0.050*	
C19	1.1582 (2)	0.6494 (3)	0.7630 (2)	0.0416 (5)	
H19	1.2119	0.7152	0.8377	0.050*	
C20	1.0191 (2)	0.6369 (3)	0.72411 (19)	0.0337 (4)	
H20	0.9789	0.6941	0.7727	0.040*	
C21	0.5393 (3)	0.7197 (4)	0.1356 (2)	0.0511 (6)	
H21A	0.6327	0.7160	0.2004	0.077*	
H21B	0.4906	0.8211	0.1538	0.077*	
H21C	0.5394	0.7398	0.0531	0.077*	

### Atomic displacement parameters (Å^2^)

**Table:**

	*U*^11^	*U*^22^	*U*^33^	*U*^12^	*U*^13^	*U*^23^
Cl	0.0278 (2)	0.0580 (3)	0.0424 (3)	0.0004 (3)	0.0107 (2)	0.0047 (3)
S	0.0401 (3)	0.0664 (4)	0.0278 (2)	−0.0108 (3)	0.0195 (2)	−0.0125 (2)
O	0.0273 (6)	0.0387 (7)	0.0259 (5)	−0.0008 (7)	0.0140 (5)	−0.0006 (6)
C1	0.0306 (9)	0.0330 (10)	0.0255 (7)	−0.0046 (8)	0.0142 (7)	−0.0049 (8)
C2	0.0298 (8)	0.0270 (9)	0.0251 (7)	−0.0008 (8)	0.0133 (7)	−0.0007 (8)
C3	0.0325 (9)	0.0265 (9)	0.0270 (7)	−0.0005 (8)	0.0158 (7)	0.0003 (8)
C4	0.0302 (8)	0.0237 (8)	0.0280 (8)	0.0014 (8)	0.0132 (7)	−0.0003 (8)
C5	0.0348 (9)	0.0347 (9)	0.0232 (7)	0.0019 (9)	0.0116 (7)	0.0025 (8)
C6	0.0352 (9)	0.0388 (10)	0.0273 (8)	0.0014 (9)	0.0178 (7)	0.0003 (9)
C7	0.0297 (8)	0.0301 (9)	0.0289 (8)	−0.0003 (9)	0.0150 (7)	0.0007 (9)
C8	0.0337 (9)	0.0278 (8)	0.0253 (7)	−0.0021 (9)	0.0134 (7)	−0.0033 (8)
C9	0.0307 (8)	0.0243 (8)	0.0305 (8)	−0.0030 (9)	0.0143 (7)	−0.0003 (8)
C10	0.0329 (10)	0.0291 (9)	0.0293 (9)	−0.0030 (8)	0.0152 (8)	−0.0034 (7)
C11	0.0324 (10)	0.0318 (9)	0.0402 (10)	0.0008 (8)	0.0209 (9)	−0.0011 (8)
C12	0.0267 (8)	0.0294 (9)	0.0321 (8)	−0.0011 (9)	0.0096 (7)	0.0049 (9)
C13	0.0336 (10)	0.0366 (10)	0.0272 (8)	0.0008 (9)	0.0099 (8)	−0.0011 (8)
C14	0.0320 (10)	0.0348 (10)	0.0321 (9)	0.0005 (8)	0.0148 (8)	−0.0034 (8)
C15	0.0306 (8)	0.0239 (8)	0.0264 (7)	0.0026 (9)	0.0118 (7)	0.0045 (8)
C16	0.0327 (10)	0.0310 (10)	0.0311 (9)	0.0025 (8)	0.0135 (8)	0.0004 (8)
C17	0.0357 (11)	0.0349 (10)	0.0428 (10)	0.0080 (9)	0.0206 (9)	0.0025 (9)
C18	0.0279 (9)	0.0415 (13)	0.0511 (12)	0.0045 (9)	0.0135 (9)	0.0003 (10)
C19	0.0337 (11)	0.0396 (12)	0.0404 (11)	0.0017 (9)	0.0050 (9)	−0.0066 (9)
C20	0.0324 (10)	0.0334 (10)	0.0328 (10)	0.0045 (9)	0.0113 (8)	−0.0030 (8)
C21	0.0511 (15)	0.0618 (15)	0.0523 (14)	0.0084 (12)	0.0334 (13)	0.0191 (12)

### Geometric parameters (Å, °)

**Table:**

Cl—C12	1.7425 (18)	C10—H10	0.9500
S—C1	1.7523 (15)	C11—C12	1.386 (3)
S—C21	1.809 (3)	C11—H11	0.9500
O—C7	1.378 (2)	C12—C13	1.382 (3)
O—C8	1.3907 (18)	C13—C14	1.390 (3)
C1—C8	1.361 (2)	C13—H13	0.9500
C1—C2	1.443 (2)	C14—H14	0.9500
C2—C7	1.390 (2)	C15—C20	1.397 (3)
C2—C3	1.401 (2)	C15—C16	1.404 (2)
C3—C4	1.394 (2)	C16—C17	1.388 (3)
C3—H3	0.9500	C16—H16	0.9500
C4—C5	1.417 (2)	C17—C18	1.382 (3)
C4—C15	1.489 (2)	C17—H17	0.9500
C5—C6	1.385 (3)	C18—C19	1.381 (3)
C5—H5	0.9500	C18—H18	0.9500
C6—C7	1.382 (2)	C19—C20	1.391 (3)
C6—H6	0.9500	C19—H19	0.9500
C8—C9	1.462 (2)	C20—H20	0.9500
C9—C10	1.396 (3)	C21—H21A	0.9800
C9—C14	1.407 (2)	C21—H21B	0.9800
C10—C11	1.384 (3)	C21—H21C	0.9800
			
C1—S—C21	99.95 (11)	C12—C11—H11	120.6
C7—O—C8	106.06 (12)	C13—C12—C11	121.37 (17)
C8—C1—C2	106.82 (14)	C13—C12—Cl	118.84 (14)
C8—C1—S	128.07 (14)	C11—C12—Cl	119.78 (14)
C2—C1—S	125.10 (13)	C12—C13—C14	119.59 (17)
C7—C2—C3	119.55 (15)	C12—C13—H13	120.2
C7—C2—C1	105.65 (15)	C14—C13—H13	120.2
C3—C2—C1	134.79 (14)	C13—C14—C9	120.33 (17)
C4—C3—C2	119.35 (14)	C13—C14—H14	119.8
C4—C3—H3	120.3	C9—C14—H14	119.8
C2—C3—H3	120.3	C20—C15—C16	117.61 (17)
C3—C4—C5	118.70 (16)	C20—C15—C4	120.92 (16)
C3—C4—C15	120.45 (14)	C16—C15—C4	121.46 (16)
C5—C4—C15	120.84 (15)	C17—C16—C15	121.11 (18)
C6—C5—C4	122.67 (16)	C17—C16—H16	119.4
C6—C5—H5	118.7	C15—C16—H16	119.4
C4—C5—H5	118.7	C18—C17—C16	120.21 (18)
C7—C6—C5	116.66 (15)	C18—C17—H17	119.9
C7—C6—H6	121.7	C16—C17—H17	119.9
C5—C6—H6	121.7	C19—C18—C17	119.66 (19)
O—C7—C6	126.34 (14)	C19—C18—H18	120.2
O—C7—C2	110.60 (14)	C17—C18—H18	120.2
C6—C7—C2	123.06 (16)	C18—C19—C20	120.46 (19)
C1—C8—O	110.86 (15)	C18—C19—H19	119.8
C1—C8—C9	134.88 (15)	C20—C19—H19	119.8
O—C8—C9	114.26 (14)	C19—C20—C15	120.92 (18)
C10—C9—C14	118.36 (17)	C19—C20—H20	119.5
C10—C9—C8	120.64 (15)	C15—C20—H20	119.5
C14—C9—C8	121.00 (16)	S—C21—H21A	109.5
C11—C10—C9	121.59 (16)	S—C21—H21B	109.5
C11—C10—H10	119.2	H21A—C21—H21B	109.5
C9—C10—H10	119.2	S—C21—H21C	109.5
C10—C11—C12	118.75 (17)	H21A—C21—H21C	109.5
C10—C11—H11	120.6	H21B—C21—H21C	109.5
			
C21—S—C1—C8	114.7 (2)	C1—C8—C9—C10	−158.1 (2)
C21—S—C1—C2	−63.9 (2)	O—C8—C9—C10	21.4 (3)
C8—C1—C2—C7	0.1 (2)	C1—C8—C9—C14	22.2 (4)
S—C1—C2—C7	178.95 (16)	O—C8—C9—C14	−158.26 (19)
C8—C1—C2—C3	−178.6 (2)	C14—C9—C10—C11	0.1 (3)
S—C1—C2—C3	0.3 (4)	C8—C9—C10—C11	−179.60 (18)
C7—C2—C3—C4	−0.9 (3)	C9—C10—C11—C12	0.6 (3)
C1—C2—C3—C4	177.6 (2)	C10—C11—C12—C13	−0.8 (3)
C2—C3—C4—C5	1.6 (3)	C10—C11—C12—Cl	178.00 (16)
C2—C3—C4—C15	−177.07 (18)	C11—C12—C13—C14	0.3 (3)
C3—C4—C5—C6	−1.3 (3)	Cl—C12—C13—C14	−178.46 (15)
C15—C4—C5—C6	177.4 (2)	C12—C13—C14—C9	0.3 (3)
C4—C5—C6—C7	0.2 (3)	C10—C9—C14—C13	−0.5 (3)
C8—O—C7—C6	178.7 (2)	C8—C9—C14—C13	179.14 (18)
C8—O—C7—C2	−0.9 (2)	C3—C4—C15—C20	150.24 (19)
C5—C6—C7—O	−179.0 (2)	C5—C4—C15—C20	−28.4 (3)
C5—C6—C7—C2	0.5 (3)	C3—C4—C15—C16	−28.6 (3)
C3—C2—C7—O	179.40 (17)	C5—C4—C15—C16	152.8 (2)
C1—C2—C7—O	0.5 (2)	C20—C15—C16—C17	−1.9 (3)
C3—C2—C7—C6	−0.2 (3)	C4—C15—C16—C17	176.97 (18)
C1—C2—C7—C6	−179.1 (2)	C15—C16—C17—C18	0.4 (3)
C2—C1—C8—O	−0.6 (3)	C16—C17—C18—C19	1.3 (3)
S—C1—C8—O	−179.45 (15)	C17—C18—C19—C20	−1.4 (3)
C2—C1—C8—C9	178.9 (2)	C18—C19—C20—C15	−0.2 (3)
S—C1—C8—C9	0.1 (4)	C16—C15—C20—C19	1.8 (3)
C7—O—C8—C1	0.9 (2)	C4—C15—C20—C19	−177.09 (19)
C7—O—C8—C9	−178.73 (16)		
